# Impact of a Semi-Rigid Knee Orthotic Intervention on Pain, Physical Activity, and Functional Capacity in Patients with Medial Knee Osteoarthritis

**DOI:** 10.3390/jcm13061535

**Published:** 2024-03-07

**Authors:** Bernd J. Stetter, Janis Fiedler, Michèle Arndt, Thorsten Stein, Stefan Sell

**Affiliations:** 1 Institute of Sports and Sports Science, Karlsruhe Institute of Technology, 76131 Karlsruhe, Germany; janis.fiedler@kit.edu (J.F.); thorsten.stein@kit.edu (T.S.); stefan.sell@kit.edu (S.S.); 2 Joint Center Black Forest, Hospital Neuenbuerg, 75305 Neuenbuerg, Germany

**Keywords:** osteoarthritis, conservative treatment, bracing, pain assessment, functional testing, activity monitoring

## Abstract

**Background**: The effectiveness of knee orthoses as part of conservative treatment for patients with medial knee osteoarthritis has not been fully explored. The purpose of this study was to evaluate the effects of a novel semi-rigid knee orthosis on pain, physical activity, and functional capacity. **Methods**: Pain levels, physical activity, and functional capacity were assessed in 24 participants experiencing symptomatic medial knee osteoarthritis one week before (i.e., pretest) initiating a six-week orthosis intervention and again during the final week of the intervention (i.e., post-test). **Results**: Night pain, pain during walking, pain during stair climbing, and pain during sitting consistently decreased by 41% to 48% while wearing the knee orthosis. Device-based measured physical activity showed a 20.2-min increase in vigorous physical activity during the post-test, while light and moderate physical activity did not show significant changes. After six weeks of orthosis application, there was a 5% increased distance for the six-minute walk test, and participants reported fewer limitations both in everyday and athletic activities, as well as an enhanced quality of life. **Conclusions**: These findings highlight the potential effectiveness of a semi-rigid knee orthosis to enhancing functional capacity and quality of life. More extensive and longer clinical trials are needed to improve confidence in these findings and understand their impact on disease progression.

## 1. Introduction

Knee osteoarthritis (KOA) is a degenerative joint disease that is prevalent worldwide and represents a major public health concern [1,2]. Medial KOA (mKOA), the most prevalent phenotype of knee osteoarthritis, is associated with the varus knee alignment (bow-legged), which primarily damages the medial aspect of the knee joint [3,4]. This alignment shifts the biomechanical loading to the medial compartment of the knee joint, leading to accelerated cartilage degeneration in that area [3,4].

KOA causes pain, disability, and a loss of physical function and, consequently, reduces the quality of life (QoL), which can stop patients from participating in home, work, or social activities [1,2,5]. Studies have also shown that KOA is associated with impaired joint proprioception [6,7]. Pain-modifying treatment and the overcoming of functional limitations are essential for preventing a downward spiral of physical inactivity and lower QoL [1,8]. An inadequate level of physical activity (PA) can result in excess bodyweight and, consequently, lead to higher mechanical joint loading and an acceleration in the degenerative process of KOA [4,9]. Furthermore, individuals with KOA are more prone to cardiovascular disease (CVD), and exercise is recommended for preventing such a disease [10]. The shared pathophysiological pathways and risk factors between CVD and KOA, such as age, hypertension, diabetes, and obesity, suggest an association between the two diseases, as indicated in a recent study by Park, Park, Ko, Choi, Min, Ahn, Kim, Koh, and Han [10], which investigated a large database (Korea National Health Insurance data). Reduced PA [11] and muscle weakness [12], prevalent in KOA patients, are also correlated with elevated CVD risk [10].

Previous studies have tried to develop strategies to encourage KOA patients to participate in greater levels of at least moderate-intensity PA [13]. PA is also known to positively affect QoL, and therefore, it is important to enable KOA patients a high flexibility in their everyday and sports activities [5]. Early treatment is pivotal and stresses the need for effective conservative treatment strategies such as exercise and the use of orthopedic aids (e.g., a knee orthosis), as surgery should be reserved for those that have not responded appropriately to less invasive methods [1].

Different types of knee orthoses (ranging from rigid braces to soft sleeves) exist [14]. Rigid braces, such as an unloader or a valgus brace, are designed as rigid shells with a hinge joint and straps to correct the lower limb force lines of the varus leg alignment to biomechanically unload the knee joint during motion [15]. The biomechanical results are encouraging, and meta-analyses on valgus bracing suggest improvements in pain [16,17], but current evidence is limited [1,15], and a lack of comfort can lead to a limited compliance [17]. Knee sleeves are a different orthotic design for the nonsurgical management of KOA, and they are typically made from elastic materials [14]. This type of orthoses does not intend to impact skeletal alignment but rather to provide joint support, mainly attributed to an improvement in proprioception by the simulation of the cutaneous mechanoreceptors [14,18]. Beneficial effects on gait and balance [19,20], pain [18,21], and functional capacity [21,22] after the use of sleeves for KOA have been reported. Another type of knee orthosis designed to strike a balance between support and flexibility is the semi-rigid knee orthosis. This orthosis combines the benefits of knee sleeves with technological advancements, such as laterally placed thin metal bars or patellar stabilization, potentially leading to increased efficacy and compliance [14]. Studies on treating ankle sprains with functional treatment have shown that the use of a semi-rigid orthosis is associated with a high level of wearing comfort and patient satisfaction [23,24]. When addressing mKOA, such an orthotic device can act as a supportive tool for both daily activities and exercise therapy, contributing to the goals of conservative mKOA treatment [25]. Dries et al. [26] concluded, based on their study on patient-reported mobility and pain, that a semi-rigid knee orthosis appears to provide suitable joint support, offering pain relief and freedom of movement. However, it is important to note that the majority of previous studies have focused on either rigid braces or soft sleeves which have been in development for a longer period [14]. Consequently, there exists a gap in the literature concerning the effectiveness of semi-rigid knee orthoses in addressing mKOA [14,25]. More specifically, there is a limited understanding of how a semi-rigid knee orthotic intervention impacts pain, physical activity, and functional capacity in individuals with mKOA, underscoring significant gaps in current knowledge.

Therefore, the aim of this study was to evaluate the effects of wearing a novel semi-rigid knee orthosis for six weeks on pain, PA, and functional capacity in mKOA patients. We expected the orthosis application to (1) reduce pain perception; (2) enhance PA; and (3) increase functional capacity.

## 2. Materials and Methods

### 2.1. Study Design and Procedure

A within-participant pretest–post-test design was employed to accomplish the aim of the study. The procedure spanned 7 weeks, with participants undergoing initial and final assessments. In the first seven days after inclusion (W0, pretest), participants were monitored without brace intervention. After this period, the participants were individually fitted with a semi-rigid knee orthosis (see knee orthosis intervention). The participants were instructed to wear the orthosis during their daily activities for 6 weeks for at least 5 h per day [25,27]. This instruction encompassed wearing the orthosis during exercise and sports activities and advised participants to remove it only for extended periods of sitting, such as desk work. During the last seven days of the six-week period, the participants were monitored with the orthosis intervention (W6, post-test). The study procedure was approved by the ethical committee of the Karlsruhe Institute of Technology. All participants gave their written informed consent prior to study participation.

### 2.2. Participants

A total of 24 patients with medial knee osteoarthritis participated in this study (Table 1). The sample was an ad hoc sample of convenience and was recruited through announcements in the local newspaper as well as via university information events concerning KOA care. Our assessment of mKOA was based on clinical and radiographic criteria (Table 2). Our evaluation of the radiological images and classification of radiological KOA signs were based on the Kellgren–Lawrence Score (K-L Score; [28]) and conducted by the same experienced orthopedist.

### 2.3. Knee Orthosis Intervention

**Figure 1:**
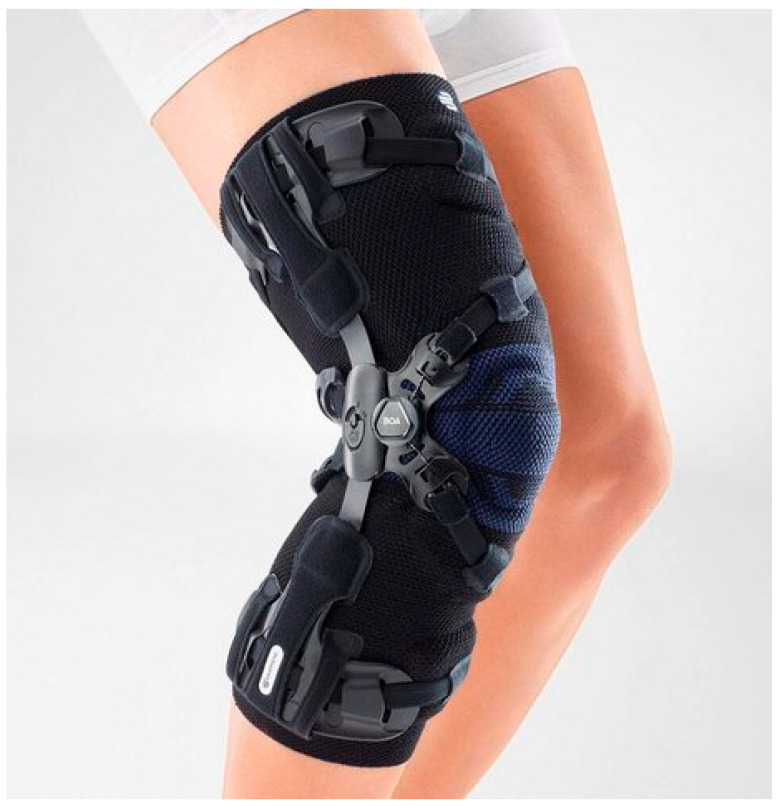
GenuTrain OA knee orthosis, ©Bauerfeind AG.

After W0, the participants were individually fitted with a semi-rigid knee orthosis (GenuTrain OA, Bauerfeind AG, Zeulenroda-Tribes, Germany) by an experienced orthopedic technician. The knee orthosis consisted of an elastic circular mesh fabric, a lateral joint splint, and an adjustable strap system following the three-point correction principle for counteracting the varus alignment (Figure 1). This orthosis is designed to relieve the knee’s painful medial compartment, promoting increased physical mobility and providing enduring comfort, particularly during extended periods of wear. An orthosis-integrated thermal sensor (Orthotimer, Rollerwerk Medical Engineering and Consulting, Balingen, Germany) was used to objectively detect wear time.

### 2.4. Data Collection

Knee pain was assessed through a daily questionnaire spanning seven consecutive days at W0 and W6. The questionnaire utilized the visual analog scale (VAS) to measure pain severity while walking, climbing stairs, sitting, and lying in bed at night [15,29]. The VAS involved a 10 cm line, ranging from “No Pain” (scored as 0) to “Worst Pain Imaginable” (scored as 10), where lower scores indicate lower levels of pain. For each VAS, the weekly average was calculated at both W0 and W6.

Estimates of PA during W0 and W6 were obtained using commercially available hip-worn (right side) activity sensors (Move 3/Move 4, Movisens GmbH, Karlsruhe, Germany). The activity sensors consisted of a 3D acceleration sensor (±16 g, 64 Hz sampling rate), a 3D rotation rate sensor (±2000 dps, 64 Hz sampling rate), a pressure sensor (300–1100 hPa, 8 Hz sampling rate), and a temperature sensor (1 Hz sampling rate). The activity data were analyzed if there was a minimum sensor wear time of at least 8 h per day for at least 4 of the 7 days during the measured weeks using epoch lengths of 10 s [30]. Non-wear time (NWT) was calculated on the sensor in 30 s intervals. The NWT detection was based on an algorithm that used accelerometry and temperature signals over a 10-min window to distinguish between wear time, NWT, and sleep [31]. The analysis software DataAnalyzer 1.13.5 (Movisens GmbH, Karlsruhe, Germany) provided by the sensor manufacturer was utilized to compute activity-related quantities, including metabolic equivalents (METs), employing established algorithms [32]. The metrics assessed in this study included NWT, steps taken, total energy expenditure (TEE), as well as durations of light PA (1.5–3.0 METs), moderate PA (3.0–6.0 METs), and vigorous PA (>6.0 METs). Daily averages were computed for steps and TEE, while the average minutes per week for light, moderate, and vigorous PA were derived by multiplying the average values per valid day by 7 to represent the total weekly minutes.

Functional capacity was assessed using the 6-min walk test (6MWT), a standardized and recommended functional assessment for KOA [33]. The covered distance offers valuable insight into the patient’s walking capacity and serves as an indicator of the individual’s endurance level [34]. The 6MWT took place in a sports hall and was conducted both at the start of W0 and at the end of W6. Two cones were positioned 20 m apart, and patients were directed to walk at their own speed, aiming to cover as much distance as possible within 6 min. Additionally, patient-reported measures were collected after W0 and W6 to evaluate the changes in the functional aspects of the knee joint due to the orthosis intervention using the validated and widely accepted Knee Injury and Osteoarthritis Outcome Score (KOOS) [35] with the following subscales: symptoms (KOOS SYM), activities of daily living (KOOS ADL), sport and leisure activities (KOOS SL), and quality of life (KOOS QoL), as well the Lequesne questionnaire [36]. The Likert scales of the analyzed KOOS subscales (with item counts varying from 4 to 17) were normalized to a scale from 0 to 100, where 0 signifies the worst outcome, and 100 denotes the best outcome. The Lequesne questionnaire generated a numerical score between 0 and 24 points (a score of 0 indicates no handicap, while scores above 14 represent an extremely severe handicap) derived from the 11 questionnaire items.

### 2.5. Statistical Analysis

All statistical tests were performed using IBM SPSS Statistics 28.0 (IBM Corporation, Armonk, NY, USA). The Shapiro–Wilk test and visual inspection of histograms were carried out to test all variables for normal distribution. At either W0, W1, or both, a significant deviation from the normal distribution was observed for all pain variables. Light PA at W0 and vigorous PA at both W0 and W1 significantly differed from the normal distribution. All functional capacity variables did not deviate from the normal distribution. As both pain and PA involved non-normally distributed variables, all comparisons between the pretest (W0) and post-test (W6) values were performed using Wilcoxon tests [21]. Functional capacity variables were tested using *t*-tests. This approach was applied to ensure consistent test usage within each category. The significance level was set a priori to 0.05. Pearson’s r was used as effect size and interpreted as r > 0.1 being a small effect, r > 0.3 a moderate effect, and r > 0.5 a large effect [37]. For the Wilcoxon tests, r was calculated by dividing the standardized test statistic value by the square root of the sample size [37]. For better comparability, Cohen’s d of the t-tests was converted to r according to Cohen [37].

Because an activity sensor was lost by one participant during W6 and due to a technical error with the sensor of one participant during W6, the activity data for two participants could not be included in the activity analysis. Three additional participants could not be included in the activity analysis as they did not meet the minimum wear time criterion for the activity sensor at either W0 or W6. Due to a technical issue, the thermal sensors did not record the orthosis wear time for two participants. Average daily wear time was calculated from all other participants.

## 3. Results

The participants wore the knee orthosis during the intervention period on average for 5.13 ± 2.95 h per day.

### 3.1. Comparison of Pain between W0 and W6

The pain levels consistently showed significantly lower scores at W6 in comparison to W0 (Table 3): Night pain decreased from 1.55 ± 2.03 to 0.88 ± 1.35 (*p* < 0.01, r = 0.64). Pain during gait decreased from 2.84 ± 1.73 to 1.54 ± 1.30 (*p* < 0.01, r = 0.83). Pain during stair walking dropped from 2.97 ± 2.07 to 1.74 ± 1.52 (*p* < 0.01, r = 0.79). Pain during sitting declined from 1.35 ± 1.40 to 0.70 ± 0.98 (*p* < 0.01, r = 0.72).

### 3.2. Comparison of PA between W0 and W6

The results for PA during the pretest and post-test periods are presented in Table 4. The duration of vigorous PA significantly increased at W6 compared to W0 (39.9 ± 38.9 min vs. 60.1 ± 52.6 min, *p* = 0.04, r = 0.47). Furthermore, the NWT showed a significant increase at W6 when compared to W0 (4698 ± 703 min vs. 5057 ± 545 min, *p* < 0.01, r = 0.63).

### 3.3. Comparison of Functional Capacity between W0 and W6

Table 5 provides an overview of the functional capacity results. The distance covered during the 6MWT significantly increased after six weeks of orthosis application (W6, 559.3 ± 73.9 m) compared to the pretest (W0, 534.8 ± 75.71 m) (*p* = 0.02; r = 0.26). All considered subscales of KOOS showed significantly higher values at W6 compared to W0 (KOOS ADL: 73.8 ± 14.7 vs. 76.8 ± 14.0, *p* = 0.01, r = 0.25; KOOS SL: 46.1 ± 27.2 vs. 53.5 ± 21.3, *p* = 0.01, r = 0.28; KOOS QoL 42.4 ± 15.3 vs. 48.1 ± 16.3, *p* < 0.01, r = 0.59) apart from KOOS SYM. Additionally, the Lequesne index showed a significantly lower score at W6 (4.1 ± 2.7) in comparison to W0 (5.4 ± 3.2) (*p* < 0.01, r = 0.30).

## 4. Discussion

This study assessed how wearing a novel semi-rigid knee orthosis for six weeks impacts pain perception, PA behavior, and functional capacity in patients with mKOA. The main findings indicate that the orthosis intervention led to (1) reduced pain intensity at night, while walking, and climbing stairs, as well as during sitting; (2) an increase in more intense activities (i.e., vigorous PA), while light and moderate PA did not show a significant change; (3) improved functional capacity, as demonstrated by an increased walking distance in the 6MWT, fewer self-reported limitations in everyday and athletic activities, and an enhanced QoL. These observations closely align with our hypothesis.

### 4.1. Effects of the Knee Orthosis on Pain

The tested knee orthosis effectively attains a crucial therapy goal by diminishing pain experienced during movement, particularly while walking; climbing stairs, often a daunting task for those with mKOA; and even during non-weight-bearing activities like sitting or at night [1]. Finger and Paulos [38] differentiated pain into night and activity pain, similar to this study, and reported a significant improvement in both aspects for wearing an unloader brace. Night pain was reduced from 3.9 to 2.6 points and pain in activity from 7.2 to 3.9 points [38]. The changes in this study are smaller, possibly linked to the lower initial pain levels noted at the start of the study: a mean value of 2.18 at the pretest, which decreased to 1.27 at the post-test. In this study, the VAS was utilized to assess pain intensity. Alghadir, Anwer, Iqbal, and Iqbal [29] demonstrated the excellent test–retest reliability and validity of this scale for application in KOA. The mean differences between W0 and W6 in all pain variables were above the reported minimum detectable change of 0.08 [29], with values exceeding 0.65.

Our findings are in accordance with the large-scale (i.e., 381 participants) patient-reported outcome measure study by Dries et al. [26], wherein the majority of participants reported a significant reduction in pain using a semi-rigid knee orthosis. Additionally, the results of this study provide further evidence for the finding of a systematic review by Cudejko, van der Esch, van der Leeden, Roorda, Parad, Bennell, Lund, and Dekker [25], who stated that soft braces can reduce pain. The daily orthosis wear times considered in the study by Cudejko et al. [25] were similar to (5 to 6 h) or even higher (up to 12 h) than those observed in this study (average daily wear time of 5.13 ± 2.95 h). The observed wearing durations can be considered to be target oriented, as pain was reduced in all investigated pain variables (i.e., night pain, pain during walking, pain during stair walking, and pain during sitting).

Overall, pain reduction in mKOA can act as a catalyst, encouraging and enabling individuals to engage in PA, which they might have otherwise avoided due to discomfort. This, in turn, leads to numerous physical, psychological, and social benefits, contributing to an improved overall QoL [1,5].

### 4.2. Effects of the Knee Orthosis on PA

The group of patients under investigation should be noted as being more physically active than the average KOA patient. This is evident as the average duration of moderate PA (W0 = 602.2 ± 228.1 min, W6 = 530.7 ± 212.7 min) exceeded the PA guidelines (>150 min/week of moderate PA), a level usually achieved by only a moderate portion of individuals with KOA [13,39].

The study’s findings indicate changes in the intensity levels of PA resulting from the use of the knee orthosis. Vigorous PA (i.e., >6.0 METs) showed a 20.2-min increase with moderate effect size. A potential reason for this could be that the knee orthosis enabled activities that might have been uncomfortable or impossible to perform without the support provided by the orthopedic aid. It is crucial to highlight that the reduced sample size (*n* = 19) limits the explanatory power in relation to this matter. Furthermore, there was a rise in NWT at W6, possibly linked with a reduction in moderate PA, although no statistically significant differences and a moderate effect size were observed for moderate PA. TEE remained unchanged between W0 and W1, indicating that the overall level of PA may not have experienced significant alterations. It should be emphasized that the observed shifts in intense PA occurred alongside a decline in pain levels. This implies that individuals with mKOA may encounter enhanced flexibility in their daily and sports activities, which could potentially have a positive impact on their overall QoL and support disease management [5,13]. This is supported by improvements observed in patient-reported measures such as KOOS ADL, KOOS SL, and KOOS QoL. Similarly, as demonstrated in the case of a knee orthosis for patellofemoral pain [40], using a knee orthosis alongside physical therapy may result in superior outcomes compared to exercising alone. However, further studies are necessary to explore the interplay between the usage of a semi-rigid orthosis, PA, and disease management.

### 4.3. Effects of the Knee Orthosis on Functional Capacity

The functional capacity, assessed using the 6MWT, demonstrates a comparable distance (pretest 559.7 ± 84.4 m), as observed in healthy individuals of similar age (60–69 years old, with a mean of 559 ± 80 m) [41]. This finding is in accordance with observations related to PA and indicates fewer functional impairments, as, for example, shown in the KOA patients prior to the surgery (323 ± 105 m) and at 12 months post total knee replacement (396 ± 112 m) [42].

Apart from the general functional status of the group of patients under investigation, a 5% increase in the distance for the 6MWT (559.3 ± 73.9 m vs. 534.8 ± 75.7 m) was observed with the application of the knee orthosis. Regarding the minimal clinically important difference for improvement, King, Hawker, Stanaitis, Woodhouse, Jones, and Waugh [42] reported a value of 74.3 m in the context of total knee replacement (baseline 6MWT distance of 323.1 ± 104.7 m), which is higher than the 29.4 m increase shown for the orthosis intervention. However, potential functional improvement is supported by KOOS QoL, which has been shown to be strongly correlated with the distance covered in the 6MWT [34].

Additional improvements were observed for KOOS ADL, KOOS SL, and the Lequesne index. These findings are supported by those of previous studies on unloading brace treatments in mKOA [27,43]. Hjartarson and Toksvig-Larsen [43] compared the effectiveness of an unloader brace with a placebo orthosis by analyzing subscales of the KOOS questionnaire at baseline and over a 52-week period. KOOS SYM, KOOS ADL, and KOOS SL improved for the brace group between 8 and 16 points [43]. Our study showed similar improvements for wearing the semi-rigid orthosis on KOOS ADL and KOOS SL, with a lower amplitude of up to seven points on average. An extended treatment duration and follow-up time may lead to greater effects. Gueugnon, Fournel, Soilly, Diaz, Baulot, Bussière, Casillas, Cherasse, Conrozier, Loeuille et al. [44] showed improvements in all KOOS subscales when participants worn an unloader brace for at least six hours a day, five days a week, over an intervention period of one year. With a mean wear time of 5.13 ± 2.95 h per day, the participants in this study achieved similar values.

Thoumie, Marty, Avouac, Pallez, Vaumousse, Pipet, Monroche, Graveleau, Bonnin, Amor, and Coudeyre [27] studied effects of an unloading knee brace on pain and functional capacity after a 6-week treatment period. They showed an average reduction of 6.0 points for the Lequesne index, which is higher than the 1.4 points observed in this study. However, the baseline level of their participants was clearly higher (13.4 ± 3.7, very severe handicap) compared to this study (5.4 ± 3.2, moderate handicap), reflecting a stronger handicap, and that more severely affected mKOA patients may potentially benefit from an orthotic support to a greater extent. Overall, a semi-rigid knee orthosis can be a helpful conservative treatment option for improving functional capacity in patients with a mild to moderate severity level of mKOA [25].

### 4.4. Limitations

This study has several important limitations that should be considered. It is worth mentioning that five participants could not be included in the analysis of PA, limiting the observed impacts of the intervention on PA behavior. Improving communication frequency during measurement periods could assist these participants in meeting the sensor wear time criteria, which was not fulfilled by three participants, thereby reducing the likelihood of application errors. It is important to note that this study considered a group of participants with a relatively low level of pain and functional disability, and consequently, the translation of the results to less active or more severely affected mKOA patients should remain speculative. Nonetheless, the findings of our study highlight pain release and functional improvement by the knee orthosis for the specific stage of early treatment in active patients. Additionally, future clinical studies may benefit from the methods used in this study and combining pain assessment, device-based PA monitoring, and functional testing. In this context, multiple assessments and the consideration of a longer intervention phase could strengthen findings. We cannot objectively confirm whether patients adhered to the orthosis usage instructions, which could introduce bias into the findings. Nonetheless, patients reported wearing the orthoses during daily activities, as well as during exercise and sports. Relying solely on announcements in newspapers and information events is also a limitation, as it might introduce a selection bias by predominantly attracting individuals who are actively interested. The absence of a control group is another limitation. However, building an actual control or placebo group is known to be difficult in such investigations due to the fact that any type of intervention can cause effects by, for example, altering proprioception [44]. In research involving orthopedic aids, subjective factors like pain perception can also be influenced by placebo effects [45]. Such effects cannot be fully excluded, as wearing the orthosis might provide participants with an enhanced sense of security or impact pain perception, even in the absence of a substantiated impact from the orthosis. Hence, additional biomechanical assessments would be valuable for substantiating the actual efficacy of the treatment in terms of relieving the medial joint compartment [17]. Finally, follow-on clinical trials and biomechanical studies are needed to confirm and understand the findings of this study in larger and diverse mKOA populations. Such studies may reveal gender-specific or KOA grade-specific effects for semi-rigid knee orthotic interventions.

## 5. Conclusions

KOA is prevalent worldwide and remains a major public health concern. The results of this study showed that wearing a semi-rigid knee orthosis as a technique for conservative treatment over six weeks potentially reduces pain perception, enhances PA, and increases functional capacity in mKOA. This knee orthosis intervention may allow individuals with mKOA to engage in activities that could have been challenging or unfeasible without the assistance provided by the orthopedic aid, thereby enhancing their functional capacity and quality of life. Additional research is required to assess whether these observations endure in more extensive and prolonged clinical trials and to determine their influence on the progression of the disease.

## Figures and Tables

**Table 1:** Characteristics of the participants at inclusion. Data are mean ± standard deviation unless otherwise indicated. K-L: Kellgren and Lawrence; KOOS: knee injury and osteoarthritis outcome score (0–100, 0, worst, to 100, best); SYM: symptoms; ADL: activities of daily living; SL: sport and leisure activities; QoL: quality of life.

Variables	Women	Men
Number	10	14
Age (years)	59.0 ± 6.2	63.1 ± 7.7
Height (m)	1.61 ± 0.08	1.76 ± 0.06
Body mass (kg)	66.3 ± 13.6	83.5 ± 11.1
Body Mass Index (kgm^−2^)	25.5 ± 4.5	27.0 ± 3.7
Involved side (left/right)	5/5	8/6
K-L Score (Grade 2/Grade 3/Grade 4)	5/4/1	1/8/5
KOOS Pain	58.6 ± 19.3	68.5 ± 13.8
KOOS SYM	62.1 ± 20.4	74.5 ± 13.1
KOOS ADL	68.9 ± 18.6	78.8 ± 10.7
KOOS SL	35.5 ± 32.4	55.7 ± 20.3
KOOS QoL	36.4 ± 17.8	45.1 ± 13.5

**Table 2:** Inclusion and exclusion criteria. mKOA: medial knee osteoarthritis; KOOS: knee injury and osteoarthritis outcome score (0–100, 0, worst, to 100, best); K-L Score: Kellgren–Lawrence; BMI: Body Mass Index.

Inclusion Criteria	Exclusion Criteria
Radiologically confirmed mKOA, i.e., K-L Score 2–4 Self-reported knee pain within the last 3 months during activities of daily living Self-reported decreased knee function Asymptomatic contralateral knee, i.e., K-L Score ≤ 2	Secondary KOA caused by trauma Contraindication of X-ray imaging BMI ≥ 35 kgm^−2^ Orthopedic injury of other joints of the lower limbs and back (e.g., rheumatoid arthritis, acute herniated disc, endoprosthesis, etc.)

**Table 3:** Pain perception (mean and standard deviation) during the pretest (W0) and post-test (W6) periods with respective *p*-values and effect sizes (Pearson’s r) as revealed by Wilcoxon tests (Wilcox). Level of significance ≤ 0.05; * marks a significant result. VAS: visual analog scale (0–10, 0, no pain, to 10, worst).

Variables	*n*	W0	W6	Wilcox	Pearson’s r
Night pain, VAS (0–10)	24	1.55 ± 2.03	0.88 ± 1.35	<0.01 *	0.64
Pain during gait, VAS (0–10)	24	2.84 ± 1.73	1.54 ± 1.30	<0.01 *	0.83
Pain during stair walking, VAS (0–10)	24	2.97 ± 2.07	1.74 ± 1.52	<0.01 *	0.79
Pain during sitting, VAS (0–10)	24	1.35 ± 1.40	0.70 ± 0.98	<0.01 *	0.72

**Table 4:** Physical activity (mean and standard deviation) during the pretest (W0) and post-test (W6) periods with respective *p*-values and effect sizes (Pearson’s r) as revealed by Wilcoxon tests (Wilcox). NWT: non-wear time; PA: physical activity; METs: metabolic equivalents. Level of significance ≤ 0.05; * marks a significant result.

Variables	*n*	W0	W6	Wilcox	Pearson’s r
NWT (min/week)	19	4698 ± 703	5057 ± 545	<0.01 *	0.63
Average daily steps	19	8209 ± 2812	7778 ± 2713	0.38	0.20
Total energy expenditure (kcal)	19	2174 ± 315	2248 ± 312	0.12	0.36
Light PA (1.5–3.0 METs) (min/week)	19	367.2 ± 164.5	338.8 ± 152.1	0.09	0.31
Moderate PA (3.0–6.0 METs) (min/week)	19	602.2 ± 228.1	530.7 ± 212.7	0.12	0.36
Vigorous PA (>6.0 METs) (min/week)	19	39.9 ± 38.9	60.1 ± 52.6	0.04 *	0.47

**Table 5:** Functional capacity (mean and standard deviation) during the pretest (W0) and post-test (W6) periods with respective *p*-values and effect sizes (Pearson’s r) as revealed by dependent *t*-tests. 6MWT: six-minute walk test; KOOS: knee injury and osteoarthritis outcome score (0–100, 0, worst, to 100, best); SYM: symptoms; ADL: activities of daily living; SL: sport and leisure activities; QoL: quality of life. Lequesne index (0–24, 0, no handicap, to 24, >14 extremely severe handicap). Level of significance ≤ 0.05; * marks a significant result.

Variables	*n*	W0	W6	*t*-test	Pearson’s r
Distance 6MWT (m)	24	534.8 ± 75.7	559.3 ± 73.9	0.02 *	0.26
KOOS SYM	24	69.3 ± 17.3	68.3 ± 18.3	0.14	0.11
KOOS ADL	24	73.8 ± 14.7	76.8 ± 14.0	0.01 *	0.25
KOOS SL	24	46.1 ± 27.2	53.5 ± 21.3	0.01 *	0.28
KOOS QoL	24	42.4 ± 15.3	48.1 ± 16.3	<0.01 *	0.59
Lequesne index	24	5.4 ± 3.2	4.1 ± 2.7	<0.01 *	0.30

## Data Availability

The raw data supporting the conclusions of this article will be made available by the authors without undue reservation.
